# Training Medical Students to Recognize, Understand, and Mitigate the Impact of Racism in a Service-Learning Course

**DOI:** 10.5888/pcd20.220367

**Published:** 2023-05-18

**Authors:** Carla Durham Walker, Gail G. McCray, Angela Wimes, David Levine, Desiree Rivers

**Affiliations:** 1 Morehouse School of Medicine, Atlanta, Georgia

## Abstract

The Morehouse School of Medicine’s Community Health Course (CHC) trains first-year medical students to work with people of racial and ethnic minorities and economically and medically disadvantaged communities. This service-learning course includes the *diagnosis/assessment* of the health of a community and the development, implementation, and evaluation of a *plan* to improve some aspect of the community’s health. The CHC teaches about the impact of racism on the health of communities through lectures, educational games, and videos focused on social determinants of health, cultural competence, and effective community engagement. Students complete small group assessments, interventions, and service activities at assigned sites. This pedagogical approach integrates the Association of Medical Colleges’ Diversity, Equity, and Inclusion competencies and engages many community partners.

The course’s strengths include a multidisciplinary faculty, a culturally and educationally diverse student body, and community partners with varied backgrounds and resources. Opportunities exist for collaborations with other degree programs to sustain and increase the impact of community interventions and link this community-based educational activity to clinical training years.

Course evaluations, exams, and short essays assess students’ awareness of racism and the extent to which unconscious bias affects students’ completion and interpretation of community assessment data and their engagement with community partners.

**Summary**

**What is already known on this topic?**

People of racial and ethnic minorities have historically received less access to quality health services, which leads to health inequities; racism is a major contributor to these inequities.

**What is added by this report?**

Although many institutions offer service-learning courses designed to train community-oriented future physicians, few provide a required, year-long competency-based course aimed at addressing the social determinants of health, particularly racism, through collaborations with communities of color.

**What are the implications for public health practice?**

Medical education plays an important role in teaching how racism affects access to and delivery of quality health care to medically underserved communities and recognizing the structures that facilitate ongoing racism in our health care system.

## Background and Rationale

The Institute of Medicine’s landmark report *Unequal Treatment* (1) concluded that “racial and ethnic minorities experience a lower quality of health services and are less likely to receive even routine medical procedures than are White Americans” (1). This report also posited that many health disparities were the result of biases and stereotypes that occur during clinical encounters, not just social determinants (1). We use the definition of racism by Dr Camara P. Jones, one of the report’s authors: **“**Racism is a system of structuring opportunity and assigning value based on the social interpretation of how one looks that unfairly disadvantages some individuals and communities, unfairly advantages other individuals and communities, and saps the strength of the whole society” (2). Health care providers with limited interaction with minority populations may exhibit some nuanced negative behaviors because of stereotypes about the lifestyle or health behaviors of their Black and Brown patients. These stereotypes may influence providers’ quality of care. In turn, patients’ health decisions might be in response to the clinician’s and staff’s behavior or past mistreatment during medical encounters. The issues identified in *Unequal Treatment* led to 21 recommendations for improvement in medical care financing, allocation of care, and the cross-cultural training of health care providers, yet the problems persist. The life expectancy of Black Americans is still 5 years fewer than that of White Americans (3). The Centers for Disease Control and Prevention estimated that in 2019, there were 70,000 premature deaths among Blacks from treatable chronic diseases. (4). This is an increase from the 1985 Secretary’s Task Force on Black and Minority Health, which estimated 60,000 excess deaths of Blacks versus Whites. This report was a major driving force for identifying solutions to health disparities and advancing health equity in the US (5). The COVID-19 pandemic both revealed and exacerbated the health disparities and health care inequities between Black and White Americans (6).

Medical academic centers and professional health organizations, including the American Medical Association, have examined their practices and developed policies to dismantle racism. They have begun to use an equity lens in hiring practices and created offices of diversity, equity, and inclusion (DEI). Additionally, anti-racism education and training of students in the health professions are recommended.

The Morehouse School of Medicine (MSM) Department of Community Health and Preventive Medicine introduced its Community Health Course (CHC) in 1998 (7). The purpose of the course is to use service-learning to train first-year medical (MD1) students to become community-oriented physicians who will provide care for diverse populations. CHC also provides instruction on ways to recognize and address racism as one of the social determinants of health (SDOH) to achieve optimal community health and health equity. This innovative course reflects the mission of MSM to provide MD1 students with the tools, skills, and self-efficacy to work comfortably with populations with which they may have had no previous experience. Further, CHC provides learners with a knowledge base of health promotion and disease prevention and control, as well as skills of community engagement, during the 2 semesters with their community partners. Competencies for the course include those outlined by the American Association of Medical Colleges (AAMC) under the domains of DEI (8). Students interact with communities and observe the reality of SDOH, including substandard housing, lack of sidewalks and transportation, and food insecurity. These examples of structural racism as a social determinant come clearly into view.

Although systemic and structural racism are often used interchangeably, each has a slightly different meaning. Systemic racism refers to entire systems, be they health care, economic, educational, or legal and includes the structures that support and maintain the systems’ race-based attributes. Systemic racism includes structural racism, which refers to the role of the structures (laws, policies, institutional practices, and entrenched norms) that support the systems (9,10). For example, historically, decisions about where major highways were constructed resulted in the destruction of communities of color, including schools and businesses. Communities that are in the shadow of these highways have higher rates of noise and air pollution and illnesses like asthma (11).

Lisa Howley, PhD, AAMC senior director of strategic initiatives and partnerships noted, “In 2018, only 40% of medical schools reported teaching about racial disparities” (12). Additionally, although SDOH have been viewed as a primary driver of health-related inequities, SDOH medical education curricular approaches have at their core under-resourcing and cultural competence instead of systems, practices, and policies that foster a focus on content rather than skills development (13–17). Thus, evolving instruction to structural-based competency in medical education is critical and produces a more substantive approach to addressing health inequities (16) — the express intent of the CHC (17,18).

The onset of the COVID-19 pandemic in 2020, intersecting with the high-profile murders of Ahmaud Arbery, Breonna Taylor, George Floyd, and others, brought to focus the roles that systemic and structural racism can play in the health outcomes of individuals and communities (19). Following these events, many publications were generated on conscious and unconscious bias in medicine and health care and recommendations on how to tackle it (19–22). Responses to the Black Lives Matter movement in the medical community include required training and continuing education among staff in medical schools and businesses and the creation of DEI offices to provide oversight and accountability. Educating medical students earlier in their training, as they are developing their ideas of what kind of physicians they will be, is imperative. Our CHC has been doing this for nearly 25 years.

The objective of this article is to describe how the CHC integrates AAMC DEI competencies into the curriculum for first-year medical students to teach the impact of racism on the health of communities and equip future physicians with skills to develop interventions to improve health outcomes.

## Course Overview

The CHC is a required first-year course for medical students that provides an interactive service-learning approach to teaching medical students about the impact of racism on the health of a community. Assessment and co-development of sustainable interventions are components.

Learners can begin to address their biases and stereotypical thinking of Black and other minority populations. Learners also address preconceived ideas in a learning environment of diverse peers who identify with populations of similar racial and ethnic backgrounds.

## Faculty and Medical Student Participants

The CHC has 25 multidisciplinary faculty consisting of physicians, nurses, health educators, public health researchers, and behavioral scientists. These faculty have devoted their careers to clinical, educational, and research endeavors that advance health equity among racial minorities and underserved populations. Faculty are provided the professional development and resources needed to reinforce course concepts and provide culturally informed instruction.

This year’s MD1 class comprises 126 racially diverse students who are divided into 12 learning communities. The MD1 class is representative of the MSM MD Program student population, which is 75% Black.

## Community Partners

**Figure 1:**
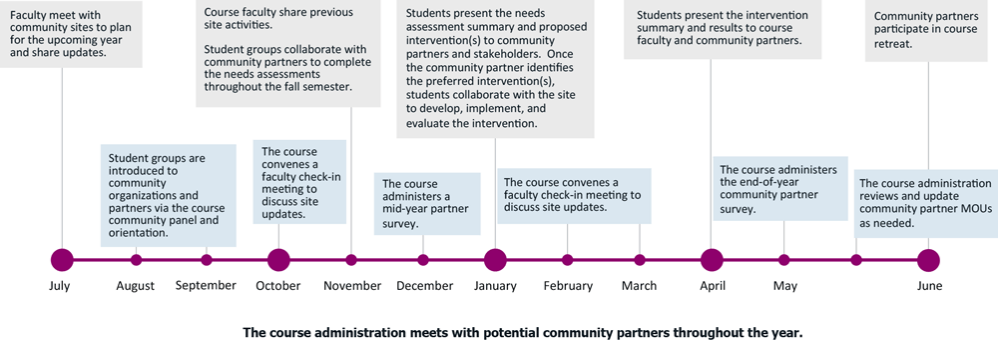
Morehouse School of Medicine Community Health Course, yearly community partner engagement timeline. Abbreviation: MOU, memorandum of understanding.

**Figure 2:**
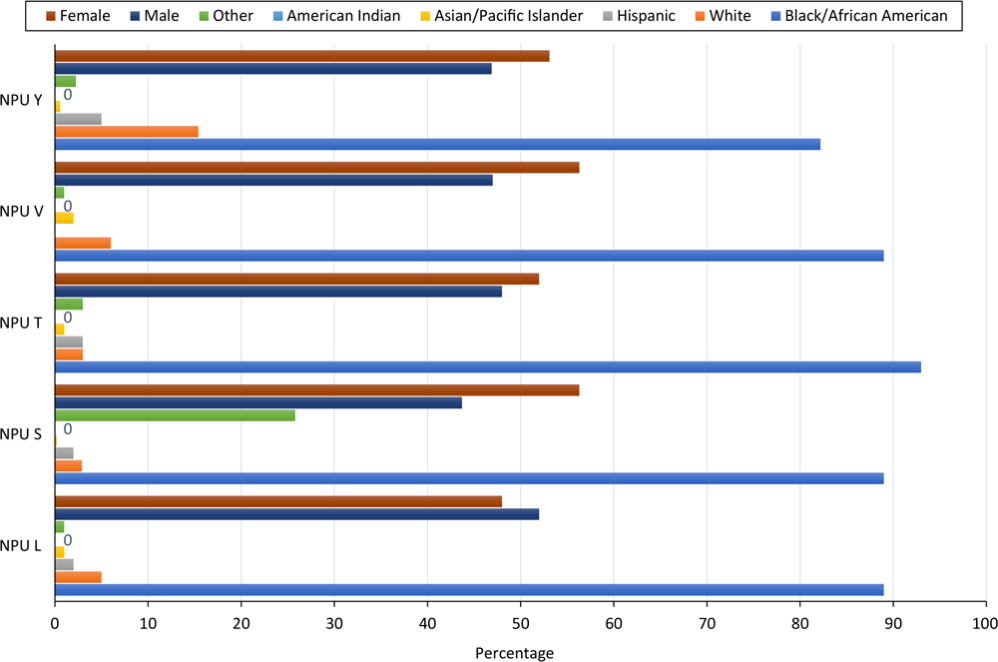
Morehouse School of Medicine Community Health Course, data on race and sex of community partners. Abbreviation: NPU, neighborhood planning unit.

Each learning community is assigned to 1 community partner site. Sites are recruited at the recommendation of the CHC faculty or by the invitation of community organizations. The CHC seeks organizations that support MSM’s mission of serving underserved communities, have staff with the time and interest to act as site liaisons, and have available space for the weekly small group meetings. Partner sites do not receive compensation for participating in the course; however, each student group receives a budget ($500 in the 2023 academic year) that is used to support community activities and provide participant incentives. Current community partners include pre-K and K-12 schools, after-school programs, independent senior living facilities, church outreach services, and a refugee social service agency in predominantly communities of color. Figure 1 illustrates the yearly timeline of the CHC engagement with its community partners. Most of the partner sites are located in Atlanta, which is divided into Neighborhood Planning Units (NPUs). NPUs were established in Atlanta to provide residents the opportunity to serve in an advisory capacity with the city government (22). Of the 5 NPU partnering sites, 82% to 93% of residents are African American (Figure 2). 

**Table:** 

NPU	Black/African American	White	Hispanic	Asian/Pacific Islander	American Indian	Other	Male	Female
L	89	5	2	1	0	1	52	48
S	89	2.9	2	0.2	0	25.8	43.7	56.3
T	93	3	3	1	0	3	48	52
V	89	6	0	2	0	1	47	56.3
Y	82.2	15.4	5	0.55	0	2.26	46.9	53.1

## CHC Overview 

### Philosophy

To help first-year medical students appreciate the similarities of “treating” individual patients and communities, a parallel is made using the clinical SOAP (Subjective, Objective, Assessment, and Plan) model (23). Akin to collecting subjective and observational data on patients, the same is collected on communities. This information is useful for assessment and diagnosis and creating a plan for treatment much like the diagnosis or assessment of the health of a community and the development and implementation of a (treatment) plan to improve some aspect of the community’s health — thus viewing the “community as patient” (18). Descriptions of the course and its curriculum components have been published previously (7,17,18).

### Curriculum

Each semester begins with an orientation and a series of large group sessions that provide foundational concepts on SDOH, cultural awareness and sensitivity, effective community engagement, community assessment, and intervention planning and evaluation. These topics are essential to preparing students to effectively engage and collaborate with their communities, with special emphasis given to teaching students to adopt a culturally aware and community-centered approach. Short essays challenge students to self-assess any biases about the community site and the surrounding NPU and to understand how SDOH, specifically racism, affect their community site and their interactions with their assigned community. Exams test students’ knowledge and application of the content presented in the large group sessions, and required readings include cultural awareness and sensitivity, effective community engagement, community assessment, and intervention planning and evaluation.

### Process

The students, in collaboration with their site contacts and with guidance from course faculty, complete a windshield survey, key informant interviews, focus groups, and optional surveys, collecting subjective and observational data to formulate a community assessment and develop an intervention. MSM students gain an appreciation of social determinants beyond biology while spending time in observation, interaction, and reviewing relevant literature about communities with similar demographics. A timeline of these activities is described in Table 1. Additionally, site representatives and community partners are important resources in understanding the assigned communities, interpreting key data during the assessments, and in developing interventions. Additionally, partner sites can use the assessment data collected by students in their efforts to pursue funding opportunities and other resources for their communities.

**Table 1:** Timeline of Students’ Course Activities, Community Health Course, Morehouse School of Medicine^a^

Timing	Activity
Pre-course	CHC orientation
Weeks 1 and 2	Students participate in large group lectures, panel discussions, and games to learn course concepts and key assessment methods.
Week 3	Students complete windshield surveys of surrounding community of the community partner sites, review previous year’s project summaries, are introduced to community site representatives, and complete their first short essay assignment.
Weeks 4–6	Students begin community service activities requested by community partner sites, plan and complete key informant interviews, and complete their second short essay assignment.
Weeks 7–9	Students complete community service activities, complete key informant interviews, take fall CHC exam, and complete their third short essay assignment.
Weeks 10–12	Students complete community service activities, plan and complete focus groups and optional survey, collect NPU and community data, and complete their fourth short essay assignment.
Weeks 13 and 14	Students complete community service activities, conclude community assessments and data collection, present draft fall presentation to community site representatives for feedback, and complete fall semester project summaries.
Week 15	Students present assessment summary and proposed intervention.
**Spring semester**	
Week 1	Students participate in large group lectures and activities to learn key intervention and evaluation planning processes.
Weeks 2–4	Students return to community sites to resume their community engagement activities, plan intervention activities, take the CHC exam, and complete their fifth short essay assignment.
Week 5	Students complete community service activities and start the community site-approved intervention projects.
Weeks 6–11	Students complete community service activities, complete and evaluate community site-approved intervention projects, and complete their sixth and seventh short essay assignments.
Week 12	Students conclude community site activities and say goodbye to community site members.
Week 13	Students present draft spring oral presentations and complete spring semester project summaries.
Week 14	Students present intervention summaries

Abbreviations: CHC, Community Health Course; NPU, neighborhood planning unit.

a Reprinted with permission from Wolters Kluwer Health, Inc.

Community partners also provide feedback through yearly surveys that assess students’ interactions with the community populations and site members and the effectiveness of their assessments, service activities, and intervention projects. The Likert scale survey questions include the following:

- Communication between me and the faculty leader(s) was effective.
- Communication with the students was effective.
- The students were well-prepared for the work they did with my community.
- The student community health assessments accurately reflect the community.
- The student projects addressed the most important needs of the community.
- As a result of taking part in this course, the health of my community has improved.
- Overall, the relationship between my site and this course has been valuable.

These surveys indicate whether the CHC community partners find the collaboration beneficial to the health of their organizations and communities. This feedback is crucial and informs the education, assessment, intervention, and engagement activities each year.

### Outcome

The student and community co-developed interventions designed to mitigate the negative impact of systemic and structural racism on the health outcomes of minority populations have included:

- Assisting low-income housing residents by developing a community garden to address nutrition needs
- Obtaining lockers for homeless shelter residents to provide more privacy
- Raising funds for bus passes for transportation to and from jobs for homeless women
- Collaborating with residents at a senior independent living facility to advocate for and maintain a traffic light and crosswalk for independence and safety
- Providing lists of local resources for childcare, housing and employment, and health assistance
- Supplementing after-school programs with health education, tutoring, and mentorship

To ensure the sustainability of these efforts, students provide summaries of their activities each year to share with subsequent classes. Throughout the CHC curriculum are clearly enumerated competencies to build anti-racism awareness and capacity among health professionals. Table 2 maps the CHC skills-based objectives with the AAMC DEI competencies, course activities, and evaluation metrics.

**Table 2:** Community Health Course Learning Objectives, Activities, and Evaluations Mapped to AAMC Diversity, Equity, and Inclusion Competencies

Skill type	Relevant AAMC DEI competency	Activity	Evaluation
**Assessment**			
Define social determinants of health	4a. Identifies systems of power, privilege, and oppression and their impacts on health outcomes (eg, white privilege, racism, sexism, heterosexism, ableism, religious oppression)	Large group lecture; small group activities; SDOH game	Exam questions; post-game discussion
Demonstrate the ability to complete a community assessment	2a. Demonstrates the value of diversity by incorporating dimensions of diversity in the patient’s health assessment and treatment plan	Small group activities	Presentation; short essay; exam questions
Use data from local, state, and federal agencies to identify a health problem	5a. Describes how stratification (eg, by race/ethnicity, primary language, socioeconomic status, LGBTQ identification) of quality measures can allow for the identification of health care disparities.	Small group activities	Group presentation
**Community Engagement**			
Demonstrate the ability to work effectively in a community setting	3a. Describes the value of working in an interprofessional team, including patients, to identify and address social risk factors influencing health (eg, food security, housing, utilities, transportation)	Small group activities	Faculty evaluation; short essays; group presentation
Demonstrate respect for addressing social determinants of health	3a. Describes the value of working in an interprofessional team, including patients, to identify and address social risk factors influencing health (eg, food security, housing, utilities, transportation)	Small group activities	Faculty evaluation; short essays; group presentation
Demonstrate sensitivity during interactions with community members	1a. Articulates how one’s own identities, power, and privileges (eg, professional hierarchy, culture, class, gender) influence interactions with patients, families, communities, and members of the health care team	Small group activities	Faculty evaluation; short essays; group presentation
Communicate effectively with those of different backgrounds, including peers, faculty, and community members	1b. Seeks and acts upon feedback regarding how one’s own identities, power, and privileges influence patients, families, communities, and members of the health care team	Small group activities	Faculty evaluation; short essays; group presentation
**Planning and Evaluation**			
Articulate the intersection between community, public, and individual health	11a. Identifies and, if appropriate, refers patients to relevant community resources that promote health equity and improve the health of local communities and populations	Large group lecture; small group activities	Exam questions
Identify and describe the components of community intervention planning, evaluation, and implementation	2a. Demonstrates the value of diversity by incorporating dimensions of diversity in the patient’s health assessment and treatment plan	Large group lecture; small group activities	Exam questions; group presentation; faculty evaluation
Effectively communicate and collaborate with community members to plan and evaluate interventions	6a. Demonstrates the practice of cultural humility and, when appropriate, provides culturally relevant resources to their patients	Small group activities	Faculty evaluation; community partner survey

Abbreviations: AAMC, American Association of Medical Colleges; LGBTQ, lesbian, gay, bisexual, transgender, and queer; SDOH, social determinants of health.

## Course Summary

The CHC has several notable strengths, weaknesses, opportunities, and threats (SWOT) in teaching first-year medical students to mitigate the impacts of racism on communities of color. These should be considered for similar course or experience development, implementation, and evaluation.

Strengths include involving a diverse, multidisciplinary, and multi-ethnic faculty, medical student body, and community that contribute to formal and informal learning and skills-building to address the impact of racism on a community’s health. Interwoven throughout CHC is the philosophy of “community as patient” and the AAMC DEI competencies that map course objectives with activities and ensure bidirectional evaluation that is beneficial to both learners and the community. A weakness of medical school instruction on racism is that knowledge and awareness can be increased, yet many factors, such as the medical school schedule, time allotted in the community, and lack of resources, may preclude a thorough assessment of the impact of racism on community health and the development of robust mitigation strategies. Medical student longitudinal rotations in the community with a commensurate evaluation of impact, as well as a commitment to sustainable interventions, are plausible solutions. Opportunities exist for collaborations with other degree programs and professionals, such as the Physician Assistant and Master of Public Health students and the Satcher Health Leadership Institute fellows, to sustain and increase the impact of community interventions. Indeed, interprofessional education is widely supported and fosters training and preparation to address racism in health and health care. Opportunity also exists to bolster the evaluation of student and course outcomes. The greatest threat to CHC is the finite community sites for which growing numbers of health professions students and degree programs seek opportunities for training. Related is the need to establish long-standing, meaningful partnerships to assess and mitigate the impact of racism. To address this, a memorandum of understanding for long-term partnerships should be established among a directory or resource list of “preferred partners.” While the effects of racism on health inequities increase, additional training is needed for health professionals to contribute to solutions.

## Implications and Next Steps

The long-term success of the course is attributed to its focus on addressing the impacts of SDOH, the long-time tenure of most of its faculty, the continued collaboration with community partners, and the support of MSM. CHC has also scaled up and evolved to accommodate increased class sizes, expanded its reach in the surrounding communities, and diversified its community partnerships to serve other vulnerable populations (ie, refugees, asylum seekers, and victims of torture). Additionally, CHC has been adopted by several of MSM’s graduate and residency programs (24–27), thereby expanding the efforts of MSM to address racism and other SDOH through various educational activities. Medical schools’ curricula could be modified to include more longitudinal community-based educational offerings. Institutions could also replicate the individual course components such as the large group lectures; small group activities via in-person, online, or flipped classroom formats; and reflective short essays. For example, the course converted its curriculum to an online format during the COVID-19 pandemic.

As CHC continues to evolve, we look forward to continuing to develop this important course, educating about racism and health inequities and developing a workforce that incorporates not only the practices and principles of community health but also advances health equity practice by addressing the needs of racially marginalized communities and others disproportionately affected by poor health and health care.

## Conclusion

Medical education can play an active role in mitigating racism and its resulting health inequities. It is important to train medical students to understand the effects of racism on the access to and delivery of quality health care and that medically underserved communities are particularly vulnerable to these effects. Medical students should also recognize the structures that facilitate ongoing racism in our health care system and be made aware of their roles and responsibilities as health care providers in this context. Medical education curricula must continue to encourage students’ self-examination and awareness of their own biases and educate them on effective strategies for advocating for disenfranchised communities and patients. 

The application of AAMC DEI competencies in the education and training of first-year medical students serves as a useful guide for medical school curricula to address the impact of racism on health care disparities in the US. When developing and implementing such curricula, it is also imperative to involve and include the perspectives of communities most impacted by racism.
